# Cultural Capital, Stigma, Class, and Hospice Care Access Among Low-Income Patients With Cancer

**DOI:** 10.1001/jamanetworkopen.2025.54797

**Published:** 2026-01-20

**Authors:** Chao Yan, Ji Ai, Tingfen Cao, Ting Jiang

**Affiliations:** 1 Nursing Department, Guizhou Aerospace Hospital (Affiliated Aerospace Hospital of Zunyi Medical University), Guizhou Zunyi, China; 2 Kiang Wu Nursing College of Macau, Macau, China; 3 Nursing Department, Third Affiliated Hospital of Zunyi Medical University, Guizhou Zunyi, China; 4 Department of Respiratory and Critical Care Medicine, Guizhou Aerospace Hospital (Affiliated Aerospace Hospital of Zunyi Medical University), Guizhou Zunyi, China

## Abstract

**Importance:**

Socioeconomic disparities in hospice care access are well recognized, but their structural drivers remain underexplored. Low-income patients with cancer often face compounded informational, psychosocial, and financial barriers at the end of life.

**Objective:**

To examine how limited cultural capital, disease-related stigma, and class-based disadvantage may be jointly associated with hospice care access among low-income patients with cancer in China.

**Design, Setting, and Participants:**

This descriptive qualitative study was conducted at a tertiary hospital in southwestern China from July 2024 to July 2025. Adult patients with advanced cancer receiving government social assistance were purposively sampled.

**Main Outcome and Measures:**

Semistructured in-depth interviews were conducted, transcribed verbatim, and thematically analyzed using a 6-phase approach. Two researchers independently coded transcripts, with analytic consensus achieved through iterative discussion. NVivo 12 software was used for data management. Analysis occurred from August to October 2025.

**Results:**

Among 16 adults with advanced cancer (median [range] age, 55 years [late 30s to early 70s]; 9 men [56.2%]), 4 themes emerged: (1) Limited cultural capital was associated with cognitive and communicative barriers, including low health literacy, reliance on informal sources, and poor digital navigation. (2) Stigma surrounding cancer and death was associated with moral dilemmas and inhibition of open discussion of hospice. (3) Economic deprivation was associated with restricted care options, reinforced curative treatment priorities, and weakened access to social support. (4) Patients and families used resilience strategies, including peer networks, communication tactics, and value redefinition. These domains interacted synergistically; poverty was associated with reduced access to education and digital literacy, with exacerbated stigma internalization and discouraged care-seeking.

**Conclusions and Relevance:**

This study found that cultural capital deficits, stigma, and socioeconomic hardship were jointly associated with a self-reinforcing cycle of hospice exclusion. These outcomes suggest that interventions must address these factors simultaneously by improving health literacy, reducing stigma, and expanding financial and systemic support.

## Key Points

**Question:**

What factors are associated with decreased access to hospice care for low-income patients with cancer in China?

**Findings:**

In this qualitative study of 16 low-income patients with advanced cancer, limited cultural capital, pervasive stigma, and socioeconomic hardship emerged as interconnected barriers that were jointly associated with restricted access to hospice care. Informal coping strategies were associated with some relief but not with overcoming structural exclusion.

**Meaning:**

These findings suggest that equitable hospice access for socioeconomically disadvantaged patients may require integrated interventions that address informational, psychosocial, and financial barriers simultaneously.

## Teaser

This qualitative study investigates the association of cultural capital, disease-related stigma, and class-based disadvantage with hospice care access among low-income patients with cancer in China.

## Introduction

Although extensive work has examined barriers to hospice access from single-disciplinary vantage points, such as economics, sociology, and culture, these approaches remain largely single axis. Economic analyses foreground affordability and payment capacity,^1^ while sociological studies emphasize information and communication asymmetries^2^ and cultural research highlights death-related attitudes and taboos.^3^ Taken in isolation, such siloed lenses obscure how economic, cultural, and psychological mechanisms coproduce barriers, and they fail to explain why patients with comparable socioeconomic status can display markedly different patterns of hospice use. The lack of an integrative framework that specifies how multiple structural forces operate synergistically has yielded a partial account of hospice exclusion, one that insufficiently addresses the systemic roots of this exclusion and offers limited guidance for comprehensive intervention design.^4^

In response, recent scholarship has called for multidimensional analyses of disparities in palliative and hospice care. However, few studies explicitly adopt such integrative perspectives. A 2023 scoping review^5^ identified a very small corpus of palliative studies using comprehensive frameworks, while a 2022 systematic review^6^ showed that patients with multiple marginalized identities (eg, racial or ethnic minority status coupled with low income) encounter compounded barriers across the cancer care continuum. Complementary quantitative evidence^7^ further indicates that intersecting axes, such as race and gender, significantly shape end-of-life care quality. Collectively, these findings underscore the need to conceptualize hospice access as an outcome associated with interlocking systems of power and exclusion, not isolated variables.

Globally, hospice and palliative care are recognized as essential to universal health coverage and improving quality of life for patients with serious illness and their families.^8^ Nonetheless, stark inequities persist; a small fraction of individuals needing palliative care receive it, revealing substantial gaps relative to Sustainable Development Goals targets. Against this backdrop, China has embedded hospice development within national health strategies (eg, Healthy China 2030) and initiated pilots to expand access.^9,10^ However, socioeconomic gradients in use remain pronounced; low-income patients with cancer systematically encounter greater obstacles to end-of-life care, reflecting entrenched health inequities.^11,12^

This study targeted 3 interrelated domains hypothesized to be associated with hospice access among low-income patients: cultural capital, stigma, and class-based disadvantage. Following Bourdieu, cultural capital comprises knowledge, language, and skills accrued through socialization that enable effective navigation of institutional fields, such as health care.^13,14^ For patients with limited education and health literacy, deficits in cultural capital translate into cognitive and communicative barriers to understanding information, engaging in shared decisions, and accessing hospice. Crucially, cultural capital is structurally distributed; unequal education and information systems generate patterned disadvantages rather than merely individual shortfalls.^15^

Disease-related stigma constitutes a second structural barrier. In many settings, including China, cancer and death are stigmatized; hospice is sometimes misread as giving up, with attendant moral pressures such as perceived violations of filial obligation.^16^ Our participants reported shame and social isolation associated with these beliefs. Stigma functions not only psychologically but also as a form of social regulation that shapes behavior.^17^ It also interacts with cultural capital; individuals with fewer informational resources are more susceptible to dominant stigmatizing narratives, while internalized stigma further deters information seeking and hospice consideration, creating a reinforcing cycle of cognitive disadvantage–stigma internalization–service avoidance.

Finally, class (socioeconomic) barriers underpin and amplify these dynamics. Financial hardship directly constrains hospice options (eg, out-of-pocket costs for palliative treatments or home care) and indirectly shapes treatment preferences and trust. Scarce resources can entrench a cure-at-all-costs orientation, rendering hospice not worth it, and poverty is linked to weaker social networks, limiting informal navigation and awareness of services. These economic, informational, and social constraints jointly hinder access for disadvantaged groups.^18^

In sum, cultural capital deficits, stigma, and socioeconomic hardship do not operate in isolation; they form an interlocking, mutually reinforcing web within health care and sociocultural contexts. We hypothesized that examining these mechanisms together would more fully explain why low-income patients with cancer struggle to access hospice. Accordingly, we conducted a qualitative study to delineate how these factors interact in association with hospice access and to elucidate the processes through which they operate, with the aim of informing multicomponent, equity-oriented strategies to expand end-of-life care access.

## Methods

### Study Design and Participants

We conducted a descriptive qualitative study to characterize barriers to hospice care among low-income patients with cancer. This design prioritized breadth of experience over phenomenological depth and used an inductive thematic approach informed by our conceptual focus.^19^ All participants volunteered to participate in the survey and signed a written informed consent form. Ethics approval was granted by Guizhou Aerospace Hospital. Reporting follows the Consolidated Criteria for Reporting Qualitative Research (COREQ) reporting guideline’s 32-item checklist for qualitative interviews or focus groups, addressing researcher characteristics, context, methods, and findings.

Participants were recruited from oncology and palliative units of a grade A tertiary hospital in Zunyi, Guizhou, China (July 2024 to July 2025). Inclusion criteria were ages 18 years or older, pathologically confirmed cancer, awareness of diagnosis, ability to communicate in Mandarin, and low-income status per government means-tested social assistance (State Council guidelines) (eAppendix 1 in Supplement 1). Exclusion criteria were severe comorbid illness, significant cognitive impairment, active psychosis, or inability to participate. Purposive sampling identified information-rich cases^20^; thematic saturation was reached by interview 13, with 3 confirmatory interviews (total, 16 interviews) (eAppendix 2 in Supplement 1).

The sample was socioeconomically homogeneous, with all participants receiving social assistance. Other intersectional dimensions (education, marital status, and cultural background) showed limited variability given that most participants had primary or junior-high education and were married, constraining identity-based analyses. Participant ethnicity was self-reported.

### Data Collection

A semistructured interview guide developed from literature and expert input covered understanding of hospice, decision processes, stigma experiences, coping, perceived informational, psychosocial, and financial barriers. Two pilot interviews refined wording and flow (not analyzed). Interviews were conducted in private rooms within the hospital or hospice ward by 2 nursing graduate students (J.A. and T.C.) trained in qualitative methods and with hospice or oncology experience. Each interview lasted 30 to 60 minutes, was audio-recorded with permission, and was accompanied by field notes on nonverbal cues and interviewer reflections.

To ensure quality, interviewers coconducted early interviews and regularly debriefed with the principal investigator (C.Y.) to align style and depth. Recordings were transcribed verbatim in Chinese, typically within 24 hours (T.C.). A second researcher (J.A.) verified each transcript against the audio; discrepancies were resolved through team discussion and, if needed, participant clarification. Transcripts were deidentified and labeled P1 through P16.

### Reflexivity and Bias Mitigation

The team maintained reflexive journals^21,22^ and held periodic meetings to examine assumptions and potential influence on interpretation; the primary interviewer was a PhD candidate with hospice experience (C.Y.). To reduce response bias, interviewers emphasized confidentiality and the absence of right and wrong answers, used neutral and open-ended prompts, and gently probed to move beyond socially desirable responses.

### Data Analysis

We applied thematic analysis in 6 phases per Braun and Clarke,^23^ supported by NVivo software version 12 (Lumivero). Two researchers (C.Y. and J.A.) independently coded transcripts line by line, combining inductive coding with coverage of a priori domains (cultural capital, stigma, and class). Codebooks were reconciled by consensus, then aggregated into candidate themes and subthemes, which were reviewed against raw data, refined, and named. The themes were (1) Familiarization with the data: repeated reading of interview transcripts to gain an overall understanding, (2) Generating initial codes: labeling and coding meaningful content in the data, (3) Searching for themes: grouping related codes to form preliminary themes, (4) Reviewing themes: assessing the coherence between themes and codes and refining the thematic structure, (5) Defining and naming themes: clarifying the essence and boundaries of each theme, and (6) Producing the report: presenting the analysis in written form.

## Results

### Participant Characteristics

A total of 16 patients participated in the study (median [range] age, 55 years [late 30s to early 70s]; 9 men [56.2%] and 7 women [43.8%]; 14 Han Chinese [87.5%] and 2 minority ethnic groups [12.5%]: 1 Miao and 1 Gelao). All participants were married and living with family caregivers at the time of the interview. Educational attainment was low; 9 participants had completed primary school, while 7 participants had a junior high school education and none had gone beyond high school. All participants were classified as low income and were receiving government social assistance; many described themselves as subsistence farmers or laid-off workers. Table 1 provides further deidentified demographics of participants.

**Table 1.** Participant Demographics (N = 16)

Patient No.^a^	Gender	Age, y	Ethnicity	Marital status	Education^b^	Diagnosis
P1	Male	40s	Han Chinese	Married	Primary school	Lung cancer
P2	Male	50s	Han Chinese	Married	Junior high school	Nasopharyngeal carcinoma
P3	Female	60s	Han Chinese	Married	Junior high school	Colorectal cancer
P4	Female	40s	Han Chinese	Married	Junior high school	Kidney cancer
P5	Female	30s	Han Chinese	Married	Primary school	Breast cancer
P6	Male	70s	Han Chinese	Married	Primary school	Prostate adenocarcinoma
P7	Female	40s	Han Chinese	Married	Junior high school	Cervical cancer
P8	Male	40s	Han Chinese	Married	Primary school	Lung cancer
P9	Male	60s	Han Chinese	Married	Junior high school	Lung cancer
P10	Female	60s	Han Chinese	Married	Primary school	Cervical cancer
P11	Male	60s	Han Chinese	Married	Junior high school	Lung cancer
P12	Male	60s	Han Chinese	Married	Primary school	Lung cancer
P13	Male	30s	Miao ethnic group	Married	Junior high school	Lung cancer
P14	Female	60s	Han Chinese	Married	Junior high school	Lung cancer
P15	Male	50s	Han Chinese	Married	Primary school	Prostate adenocarcinoma
P16	Female	40s	Gelao ethnic group	Married	Primary school	Breast cancer

^a^ All names are coded.

^b^ Primary school indicates completion of elementary education (approximately sixth grade). Junior high indicates completion of middle school (approximately ninth grade).

Despite the relatively small sample size, we achieved a diverse representation of cancer diagnoses and both genders. However, the group was homogeneously low income and predominantly of similar educational and cultural background, which is a limitation in capturing a wider range of social experiences.

### Overview of Themes

Through inductive analysis of interview transcripts, we identified 4 core themes, each with several subthemes, that together illustrate the barriers to hospice care access among low-income patients with cancer. These themes and subthemes are summarized subsequently and described in detail, with exemplar quotations. See Table 2 for details.

**Table 2.** Interview topics

Themes	Subthemes
Lack of cultural capital and multidimensional cognitive barriers	Difficulties in decoding and understanding information
	Reliance on informal information channels
	Insufficient patient-clinician communication skills
	Digital health literacy gap
Generation, internalization, and individual coping of stigma	Social construction and perception of stigma
	Moral dilemmas and decision-making silence
	Psychological defense and meaning reconstruction
Resource deprivation and opportunity constraints under class barriers	Absolute limitations in financial capacity
	Entrenched cure-oriented attitudes
	Weak social capital and failure in resource mobilization
Resilience building and resistance practices under structural exclusion	Creation of informal support systems
	Strategic communication and negotiation
	Reanchoring of value systems

Notably, these themes are interconnected rather than independent. Participants often described experiences in which multiple factors were at play simultaneously (for instance, how financial hardship exacerbated their information gaps, or how stigma and low literacy combined to deter them from asking about hospice). We present each theme separately for clarity, but we also highlight intersections where relevant. Participants are referred to by their coded identifiers (P1 through P16), along with relevant characteristics, such as gender, age (in decades for deidentification), and education level to provide context to quotes.

#### Theme 1: Lack of Cultural Capital and Multidimensional Cognitive Barriers

Deficits in cultural capital manifested as systematic cognitive and communication barriers. These constituted the primary obstacle to accessing hospice care.

##### Information Decoding and Comprehension Barriers

Most interviewees demonstrated significant difficulty understanding professional terms, such as “hospice care” and “palliative treatment,” often equating them with “giving up treatment” or “waiting to die.” P5 (female, 30s, primary school education) stated, “When the doctor said ‘palliative treatment,’ we didn’t understand. We thought it meant there was no hope left and we should just go home and wait.” This chasm between specialized terminology and everyday language led to fundamental misunderstandings and avoidance of services.

##### Reliance on Informal Information Channels

Limited by educational attainment and social networks, respondents heavily relied on informal channels for information. Nonprofessional online articles, short videos, and word-of-mouth communication among patients served as the main sources of information. P2 (male, 50s, junior high education) said, “I just listen to what others in the ward say or whatever I come across online. I don’t know whose word to trust.” This reliance exacerbated information fragmentation, inaccuracy, and the risk of misinformation.

##### Inadequate Clinician-Provider Communication Skills

Interviewees generally struggled to communicate effectively with health care professionals. They found it difficult to clearly express their symptoms and psychological needs and also to conduct in-depth inquiry and negotiation during decision-making processes. P8 (male, 40s, primary school education) said, “The doctor spoke very fast and used many terms I’d never heard. I felt too embarrassed to keep asking, so I just nodded.” This dynamic placed patients in a passive, vulnerable position within the patient-clinician relationship.

##### Digital Health Literacy Gap

In the digital era, low cultural capital has engendered a new disadvantage: the digital health literacy gap. Despite the availability of various health apps and online platforms, respondents faced difficulties characterized by not knowing how to use them, being afraid to use them, and being unable to use them effectively. P16 (female, 40s, primary school education) said, “My child downloaded a hospital app for me, but it was full of options. I couldn’t find what I needed, was afraid of clicking wrongly, and eventually stopped using it.”

This digital divide excluded patients from increasingly digitized medical information services. P11 (male, 60s, junior high education) remarked, “I’ve heard people say that artificial intelligence [AI] can diagnose illnesses better than doctors. I wonder if AI could offer new options for my condition.”

#### Theme 2: Generation, Internalization, and Individual Coping of Stigma

Stigma is not a static psychological state but a dynamic process. It is socially constructed and internalized and subsequently associated with behavior.

##### Social Construction and Perception of Stigma

Cancer is widely labeled as an incurable disease and a symbol of misfortune, while seeking hospice care is often misinterpreted as passively waiting for death and a lack of filial piety. Interviewees were acutely aware of such social prejudices. P10 (female, 60s, primary school education) remarked, “The neighbors started avoiding us after they found out. I’m sure they were talking behind our backs, saying this family has a bad illness.” This perception of exclusion is a core source of stigma.

##### Moral Dilemmas and Decision-Making Silence

Under the dual pressure of social stigma and filial piety culture, family decision-making was trapped in profound moral dilemmas. Opting for hospice care risked exposing families to moral condemnation for “abandoning a loved one.” As a result, many families chose decision-making silence, avoiding discussion and decisive action. P11 (male, 60s, junior high education) reflected on this dilemma, stating, “We can’t stop treatment. Otherwise, my children would be condemned by others. No matter how hard it is, we have to keep going.”

##### Psychological Defense and Meaning Reconstruction

To cope with intense stigma, some patients and families developed various psychological defense mechanisms. Some adopted a strategy of denial, refusing to acknowledge disease progression; others engaged in meaning reconstruction, redefining the value of end-of-life experiences. After a period of anguish, P3 (female, 60s, junior high education) reflected, “Eventually, I came to terms with the reality. If a cure isn’t possible, at least ease my pain. Accompanying my family peacefully through the final days—that’s also a way of fulfilling our duty to the family.” This reframing represents a subtle yet significant resistance to dominant stigmatizing narratives.

#### Theme 3: Resource Deprivation and Opportunity Constraints Under Class Barriers

Economically disadvantaged status systematically translates into deprivation of resource access opportunities. Outcomes extend far beyond payment capacity alone.

##### Absolute Constraints on Financial Capacity

This constitutes the most direct and brutal barrier. Many effective but self-paid pain-management medications and professional home care services were excluded from consideration due to unaffordability. P4 (female, 40s, junior high education) stated tearfully, “The painkiller the doctor recommended costs hundreds of yuan per day. We can’t afford it. I just have to endure the pain.” P14 (female, 60s, junior high education) added, “The doctor told me to maintain my PICC [peripherally inserted central catheter] line regularly at the community hospital after discharge, but our local hospital sometimes doesn’t allow reimbursement. Going to the city hospital is both time consuming and expensive.”

##### Entrenchment of Cure-Oriented Mindsets

Economic insecurity paradoxically reinforced an obsession with complete cure. Limited funds must be used on what is perceived as truly effective (ie, treatments that may cure). Hospice care, which improves quality of life without extending survival, was deemed “not worth the investment.” P15 (male, 50s, primary school education) expressed a typical view, saying, “Money must be saved for chemotherapy. As for pain relief, we can just endure it.”

##### Weak Social Capital and Failure in Resource Mobilization

Low income implies homogenized and scarce social networks. These individuals’ circles similarly lacked valuable medical information and resources, unable to provide critical support, such as knowing a certain expert or understanding specific policies. P13 (male, 30s, junior high education, Miao ethnicity) articulated this dilemma, saying, “We don’t know anyone in the city, no idea where to ask or what subsidies we qualify for.” Institutional resources existed in theory but remained inaccessible due to deficient social capital.

#### Theme 4: Resilience Building and Resistance Practices Under Structural Exclusion

Despite facing multiple structural oppressions, interviewees did not remain entirely passive. Instead, they demonstrated agency by constructing resilience and engaging in subtle resistance through various strategies.

##### Creation of Informal Support Systems

When formal support systems failed, patient families turned to building informal support networks. Peer support groups and hometown associations became crucial spaces for exchanging information, sharing experiences, and providing mutual emotional support. P7 (female, 40s, junior high education) noted, “Joining a patient group made things a bit easier. Everyone is struggling alike. We tell each other which doctors are good, how to get reimbursements. It gives some peace of mind.” P12 (male, 60s, primary school education) said, “I follow the community worker’s recommendation to try traditional Chinese medicine. It’s cheaper and doesn’t require injections.”

##### Strategic Communication and Negotiation

Some families developed tactics for engaging with the system. For instance, interviewees mentioned having the most educated child communicate with doctors or employing strategies of showing vulnerability and pleading to secure more attention and explanations from medical staff. This represents a form of survival wisdom within power asymmetries. P1 (male, 40s, primary school education) shared, “I always bring my son when discussing my condition with the doctor. He’s a college student. He understands more than I do.” P4 (female, 40s, junior high education) explained, “When the doctor recommends treatment options, I don’t know what suits me. I always explain my family’s financial difficulties and ask if there are domestic drugs or alternative treatments.”

##### Reanchoring of Value Systems

Through prolonged treatment experiences, some families underwent a value shift from cure to comfort. They began redefining what doing right by the patient meant, transitioning from pursuing survival at all costs to cherishing present companionship and alleviating suffering. P9 (male, 60s, junior high education) ultimately decided, “I’m done with fighting this illness. I choose to go home, rest properly, and let nature take its course, surrounded by my family as I complete life’s journey.” P6 (male, 70s, primary school education) concluded, “I’m already 70. Every treatment leaves me feeling worse. Since it’s late stage, I’d rather return home early, eat and drink what I want, live comfortably, and stop tormenting myself.” This reanchoring of values constitutes an adaptive resistance to dominant medical culture and reflects intrinsic resilience.

## Discussion

### Interlocking Barriers and a Cyclical Mechanism

In this qualitative study, we found that barriers to hospice care access for low-income patients with cancer in China were associated with interlocking factors of cultural capital deficits, stigma, and class-based disadvantages. These factors formed a mutually reinforcing system that perpetuated structural exclusion. Our findings revealed a cyclical mechanism: economic poverty was associated with restricted cultural capital accumulation, which was associated with increased risk of internalized stigma; internalized stigma then rationalized socioeconomic disadvantage, which was associated with avoidance or unawareness of hospice. This synergy underscores that solutions need to be multifaceted, moving beyond single-dimensional interventions.

### Theoretical Interpretations and Extensions

#### Cultural Capital in the Digital Age: The Dual Exclusion

The digital health literacy gap extends Bourdieu’s account by showing that low-income patients now face dual exclusion, from both traditional medical communication and digitized health platforms. As health care embraces telehealth and AI, this gap risks widening disparities.^24,25^ Future technology integration could therefore be coupled with accessible education and user-centered design to avoid exacerbating inequities.

#### Stigma as Moral Discipline: The Role of Filial Piety

Our findings indicated that stigma operated as a dynamic process of social construction, internalization, and action. A critical finding was that filial piety could be co-opted as a form of moral discipline, pressuring families into decision-making silence or excessive treatment. This adds a culturally specific dimension to stigma theory, showing how revered values can become barriers. Interventions could reframe hospice as consistent with, not contrary to, familial devotion.

#### Agency and Resilience: Tactics of Individuals With Less Agency

The theme of resilience highlighted the agency and resourcefulness of patients. Through informal support, strategic communication, and value reanchoring, participants enacted what de Certeau termed “tactics of the weak,” ^26^ or tactics of individuals initially lacking agency. Acknowledging these practices is vital, shifting the narrative from victimhood to partnership. Future interventions could amplify these grassroots strategies through codesign and community-based support.

### Implications for Policy and Practice

To address these intertwined barriers, integrated interventions are essential. We recommend the following:

1. Pairing financial subsidies with patient navigation services to bridge cultural and informational gaps.
2. Launching public campaigns led by trusted community figures to destigmatize hospice care and reframe it as a compassionate choice.
3. Implementing structurally sensitive reforms in health care, including training clinicians to recognize nonclinical barriers and simplifying access to social assistance.

These insights also inform global health equity efforts, emphasizing that expanding palliative care under universal health coverage must be attuned to local sociocultural contexts. These recommendations align with World Health Organization guidance to integrate palliative care into primary health care and with Sustainable Development Goal 3.8 on universal health coverage.

### Limitations

This study has several limitations. The single-site, socioeconomically homogeneous sample limits the transferability of findings to more diverse populations, including those from rural areas, middle-income groups, or differing cultural and religious backgrounds. The lack of demographic variation also constrains exploration of identity-based intersectionality. While participants demonstrated notable resilience strategies, the design precludes assessment of long-term associated outcomes. Future longitudinal and interventional studies are needed to evaluate whether such coping practices are associated with improved hospice uptake, symptom management, or end-of-life quality. As with all qualitative research, findings are context specific; although themes may be conceptually transferable, their expression may vary across cultural or health system contexts.

## Conclusions

Through in-depth qualitative analysis, this study revealed that low-income patients with cancer in China faced multidimensional barriers to hospice care rooted in the complex, synergistic interplay of cultural capital deficits, disease-related stigma, and class-based disadvantage. These factors did not operate in isolation but instead constituted a self-reinforcing network of structural exclusion that was systematically associated with limited access to appropriate end-of-life services.

The findings suggest that no single-dimension intervention, such as financial subsidies or antistigma campaigns alone, is sufficient to disrupt this cycle. Advancing equitable hospice access will require integrated strategies that simultaneously promote cultural empowerment (eg, enhance health literacy and communication capacity), implement destigmatization efforts (eg, reshape societal narratives about hospice care), and coordinate structural support (eg, expand insurance coverage and social safety nets). Such multifaceted approaches are essential to dismantle systemic barriers and advance end-of-life health equity in alignment with core principles of universal health coverage.

## Notes

Supplement 1. **eAppendix 1.** Criteria for Defining Low-Income Groups in China **eAppendix 2.** Interview Outline

Supplement 2.
Data Sharing Statement
