# Supplementary material for: Cultural Capital, Stigma, Class, and Hospice Care Access Among Low-Income Patients With Cancer
Source: JAMA Netw Open. 2026 Jan 20;9(1):e2554797. doi: 10.1001/jamanetworkopen.2025.54797 (PMC12820735; doi:10.1001/jamanetworkopen.2025.54797)
Supplement: Supplement 1. — eAppendix 1. Criteria for Defining Low-Income Groups in China eAppendix 2. Interview Outline [file jamanetwopen-e2554797-s001.pdf]

Supplemental Online Content

Yan C, Ai J, Cao T, Jiang T. Cultural capital, stigma, class, and hospice care access among low-income patients with cancer. *JAMA Netw Open*. 2026;9(1):e2554797. doi:10.1001/jamanetworkopen.2025.54797

eAppendix 1. Criteria for Defining Low-Income Groups in China
eAppendix 2. Interview Outline

This supplemental material has been provided by the authors to give readers additional information about their work.

© 2026 Yan C et al. *JAMA Network Open*.

Criteria for Defining Low-Income Groups in China

The criteria for defining low-income groups in China are as follows:

I.Minimum Living Security Recipients: Households whose per capita income is below the local minimum living security standard and whose property status meets the specified requirements.

2. People in Extreme Difficulty: Individuals such as the elderly and persons with disabilities who have no ability to work, no source of income, and no statutory supporters or dependents.

3. Monitoring Objects for Preventing Return to Poverty: Rural households that still face the risk of returning to poverty after being lifted out of poverty.

4. Households on the Verge of Minimum Living Security: Households whose income is below I.5 times the local minimum living security standard (with some regions allowing a relaxation to 2 times) and whose property status meets the specified requirements.

5. Households with Difficulties in Rigid Expenditures: Households whose income is below the local per capita disposable income of residents, and the proportion of necessary expenditures such as medical care and education exceeds the locally specified ratio.

© 2026 Yan C et al. *JAMA Network Open*.

Interview Outline

I. Disease Course and Decision-Making: Please describe the treatment process of yourself/your family member after diagnosis, as well as the decision-making process regarding major treatment choices (e.g., hospital transfer, adjustment of treatment plans, selection of palliative care).

2. Information Acquisition, Understanding, and Barriers: Through which channels did you learn about "hospice care" or "palliative care"? What was the greatest difficulty you encountered in the process of understanding these concepts?

3. Economic Impact and Specific Examples: To what extent did costs influence your selection of treatment and care plans? Could you provide specific examples to illustrate this?

4. Social Perceptions and Internal Feelings: What do you think people around you perceive of "advanced cancer" and "not pursuing cure-oriented treatment"? Have you experienced pressure or felt the need to conceal relevant situations due to these perceptions?

5. Doctor-Patient Communication Experience: Was your communication with doctors smooth? Have you ever encountered difficulties in expressing your needs or understanding medical advice? Which patients/family members do you think are more likely to receive attention from doctors?

6. Intersection of Identity and Predicaments: As a family with a specific background (e.g., financially disadvantaged/rural households), did you encounter any unique difficulties when seeking care?

7. Needs and Expectations: During the most difficult period, what kind of support system did you hope to have to assist you and your family?

8. Additional Comments: Are there any other important experiences or feelings that you consider significant but have not been covered in the above questions?

© 2026 Yan C et al. *JAMA Network Open*.
