# Supplementary material for: Cultural Capital, Stigma, Class, and Hospice Care Access Among Low-Income Patients With Cancer
Source: JAMA Netw Open. 2026 Jan 20;9(1):e2554797. doi: 10.1001/jamanetworkopen.2025.54797 (PMC12820735; doi:10.1001/jamanetworkopen.2025.54797)
Supplement: Supplement 2. — Data Sharing Statement [file jamanetwopen-e2554797-s002.pdf]

Data Sharing Statement

Yan. Cultural Capital, Stigma, Class, and Hospice Care Access Among Low-Income Patients With Cancer. *JAMA Netw Open*. Published online January 20, 2026. doi:10.1001/jamanetworkopen.2025.54797

Data
Data available: No

Additional Information
Explanation for why data not available: The datasets generated during and analysed during the current study are available from the corresponding author on reasonable request.
